# Microfluidics-assisted electrospinning of aligned nanofibers for modeling intestine barriers

**DOI:** 10.7717/peerj.13513

**Published:** 2022-06-07

**Authors:** Wentao Su, Miao Zhang, Wenbo Wei, Haitao Wang, Wei Zhang, Zhongyu Li, Mingqian Tan, Zongzheng Chen

**Affiliations:** 1 School of Food Science and Technology, National Engineering Research Center of Seafood, Dalian Polytechnic University, Dalian, Liaoning, China; 2 Academy of Food Interdisciplinary Science, Dalian Polytechnic University, Dalian, Liaoning, China; 3 The First Affiliated Hospital of Shenzhen University, Shenzhen Second People's Hospital, Shenzhen, China; 4 Research Center for Clinical Pharmacology, Southern Medical University, Guangzhou, China; 5 Dalian Minzu University, Dalian, China

**Keywords:** Microfluidics, Foodborne nanofibers, Electrospinning, Nanofiber alignment, Intestine barriers

## Abstract

During electrospinning, the fibers deposited on the collector are usually randomly oriented in a disordered form. Researchers hope to generate periodic structures to expand the application of electrospinning, including improving the sensing properties of electronic and photonic devices, improving the mechanical properties of solid polymer composites and directional growth of human tissues. Here, we propose a technique to control the preparation of aligned foodborne nanofibers by placing dielectric polymers on microfluidic devices, which does not require the use of metal collectors. This study was conducted by introduced PEDOT:PSS polymer as a ground collector to prepare aligned foodborne nanofibers directly on the microfluidic platform. The fluidity of the electrolytic polymer collector makes it possible to shape the grounding collector according to the shape of the microcavity, thus forming a space adjustable nanofiber membrane with a controllable body. The simplicity of dismantling the collector also enables it extremely simple to obtain a complete electrospun fiber membrane without any additional steps. In addition, nanofibers can be easily stacked into a multi-layer structure with controllable hierarchical structures. The Caco-2 cells that grow on the device formed a compact intestinal epithelial layer that continuously expresses the tightly bound protein ZO-1. This intestinal barrier, which selectively filters small molecules, has a higher level of TEER, reproducing intestinal filtration functions similar to those of *in vivo* models. This method provides new opportunities for the design and manufacture of various tissue scaffolds, photonic and electronic sensors.

## Introduction

Nanofibers can mimic the structure of the extracellular matrix structure due to their high specific surface area, which is a research focus in the field of biomedicine and biomaterials. Organized various types of nanofiber structures controllable and orderly is one of the crucial challenges in this field. Electrospinning is a simple method that can control the layout of nanofibers. This technology makes it possible to prepare nanofibers with diameters from 10 nanometers to several microns simply and multifunction by selecting a variety of materials, including metals, ceramics, synthetic and natural polymers (Naghdi et al., 2020; Xue et al., 2017; Shan, Li & Huang, 2020). For example, polyvinylpyrrolidone (PVP) is widely used in the preparation of materials containing functional nanofibers of pharmaceutical nanofibers, inorganic organic composites and liposomes due to its remarkable properties, such as high hydrophilicity, complexing capacity, biocompatibility (Biswas et al., 2006; Li et al., 2022). Although functional fibers are widely used in materials science, such as many standard processes established in the food industry, they are still less used in the food production and engineering. Nanofibers can be used to regulate the sensory and physicochemical properties of delicious foods, such as texture, flavor, appearance and stability (Hada & Goli, 2019; Aytac et al., 2017; Alehosseini et al., 2019; Shababdoust et al., 2020). Electrospinning nanofibers can also be suitable packaging materials, used to package and protect bioactive ingredients from external environmental factors, and has been used as an ideal platform for health food packaging and spoiled food monitoring (Aytac et al., 2017; Alehosseini et al., 2019; Choi et al., 2020; Lin, Ni & Pang, 2019; Lin et al., 2019).

Microfluidic devices, also known as lab-on-chips, are characterized by the flexible combination and scale integration of various cell technologies on the micro controllable platform (Whitesides, 2006; Su, Liang & Tan, 2021a; Su, Liang & Tan, 2021b). The platform can accurately control and manipulate the microscale fluid, especially suitable for preparing electrospun nanofibers with specific patterns and arrangements (Hu et al., 2017). In a typical process, electrospun nanofibers based on microfluidics are collected on the grounded metal electrode. Due to the bending instability of the high charge jet, electrospun fibers are usually deposited on the surface of the electrode plate randomly to form two-dimensional (2D) randomly oriented nanofiber mats (Reneker et al., 2000; Park et al., 2018). Park & Kim (2015) prepared spatially patterned and disordered nanofiber mats on the surface of potassium chloride solution using microfluidics-assisted electrospinning technology. In addition to the above disordered nanofiber mats, electrospun nanofibers with specific patterns and arrangements have attracted increasing attention due to their outstanding potential in creating functional structures or devices. For example, the spatial arrangement of nanofibers can induce anisotropic optical polarization (Yao et al., 2007), and provide guidance clues for cell arrangement, differentiation and migration through external internal signals (Hu et al., 2017; Li et al., 2019; Chen et al., 2019). The arrangement and pattern making of electrospun nanofibers able to create advanced products, such as multifunctional sensors (Guo & Ding, 2021) and cell culture media with micro/nano hybrid structure (Park et al., 2018; Jia et al., 2014). However, the electrospun materials used in the above literature need to be dissolved with additional toxic chemicals, which limits their applications in bioengineering, health care and food industry. Furthermore, the adhesion between the electrospun nanofiber felt and the metal surface is very strong, which will lead to the morphological change of the nanofiber felt in the stripping process. Therefore, it is ideal for developing a new method to generate well-aligned food-grade nanofibers and to deposit or transfer them to the surface of the solid substrate for device manufacturing.

In this article, we propose a novel method for the preparation of edible aligned fibers based on microfluidic devices simply, which needn’t to use metal collectors and toxic chemicals. This study introduced patterned PEDOT:PSS polymer as a ground collector to prepare ordered nanofibers directly on the microfluidic platform. The fluidity of the electrolytic polymer collector makes it possible to shape the grounding collector according to the shape of the microcavity, thus forming a space controllable nanofiber membrane with a complex structure. The simplicity of dismantling the collector also enables it extremely simple to obtain a complete electrospun fiber membrane independently without any additional steps. In addition, nanofibers can be easily stacked into a multilayer structure with controllable hierarchical structure for modeling intestine barriers This method provides further opportunities for the design and manufacture of new health food customization, tissue scaffolds, photon and electronic sensors.

## Materials and Methods

### Materials

Poly(3,4-ethylenedioxythiophene)-poly(styrenesulfonate) (PEDOT:PSS) was provided by aladdin as a surfactant-free aqueous dispersion with 1.5 wt% in deionized water. Polyvinylpyrrolidone (PVP) (1,300 kDa, Macklin), Zein (99.5%, Shandong Jiuyu Biology Development Co. Ltd, Shandong, China), β-Cyclodextrin (98%, Macklin), Photoresist (SU-8 3035; MicroChem Co. Ltd, Euless, TX, USA), SYLGARD™ 184 silicone elastomer kit (Dow Corning Co. Ltd, Midland, MI, USA).

### Preparation of microfluidic devices *via* soft lithography

Microfluidic device was fabricated by the standard soft lithography method we previously reported (Jiang et al., 2012). In short, a clean silicon wafer was first spin coated with a negative photoresist (Microchem Co. Ltd, Euless, TX, USA). After the photoresist is baked at 95 °C for 20 min, the resist is exposed to ultraviolet light through a photomask made by CAD software, and developed in the developer 2-Acetoxy-1-methoxypropane (Aladdin Co. Ltd, Shanghai, China) solution. Then, PDMS was poured into the silicon wafer and cured on a hot plate at 80 °C. Finally, the replica of the microfluidic device is stripped from the silicon wafer.

### Electrospinning of aligned foodborne nanofibers on microfluidic devices

In this study, the electrospinning equipment used which included high pressure supply units, pinning nozzle, syringe pump and collection unit (Jiang et al., 2012). The polymer solution containing 5–20 wt% PVP or zein solution were prepared by using anhydrous ethanol as solvent in a certain volume ratio for electrospinning. β-cyclodextrin solution was prepared for electrospinning at the concentration of 180 wt% in deionized water as reported by Celebioglu & Uyar (2020). The distance between the syringe needle and the collector is about 12 cm, and the applied voltage is set at 13 kV. PVP, zein and hydroxypropyl-beta-cyclodextrin (HP-β-CyD) solutions were continuously supplied to the needle by a syringe pump at a certain flow rate, and the electrospinning time was usually 1 min. Humidity maintained at 75–85% in the whole experiments. The highly concentrated (180%, w/v) of HP-β-CyD were prepared in distilled water (Celebioglu & Uyar, 2020), while zein (20%, w/v) were prepared in ethanol/water with the ratio of 70:30 (v/v) (Yao, Li & Song, 2007).

A microfluidic device with microchannel and microcavity was fabricated by using the soft lithography technology. The surface of microchannel was modified by plasma treatment (300 W, 20 s) to obtain hydrophilic surface. A conductive solution is added at inlet of the microchannel (PEDOT:PSS), and the electrolyte solution was evenly distributed on the hydrophilic pattern on count of the wetting characteristics of the microchannel. Then drying for 24 h, which enable to establish the position-selective patterning of the electrolyte solution in microfluidic device. Next, high voltage is applied between the metal needle and the patterned electrode on the microfluidic device for electrospinning, and the aligned nanofiber mats are generated on the surface of the dielectric patterned electrode. As the control experiment, copper strips was used to collect the nanofibers.

### Morphological characterization of intestinal barrier

Human colorectal cells (Caco-2, Cell Bank of the Chinese Academy of Sciences, Shanghai, China) were cultured in high glucose DMEM medium containing 10% fetal bovine serum and 1% penicillin-streptomycin solution at 37 °C and 5% CO_2_. After the preparation of the chip, ultraviolet disinfection was carried out for 2 h before the start of the experiment. The hose was connected to the chip and the DMEM medium was perfused overnight. Finally, the Caco-2 cells were digested with trypsin and made into a mixed suspension, and the cell suspension with a density of 1 × 10^6^ cell/mL was inoculated into the channel. After the cells were settled for 2 h, the microchip was placed in a cell incubator for 24 h until the cells grew to form an intact intestinal barrier. The cell viability was detected by CCK-8 method, that is, 10 μL CCK-8 reagent and 90 μL cell culture medium were added into the chip channel, and the absorbance at 450 nm was measured by microplate reader after 2 h of incubation (Li et al., 2022).

The resulting intestinal barrier was subsequently morphologically characterized by immunohistochemistry. To assess tight junctions between cells at the top of the intestinal barrier, confocal immunofluorescence microscopy was used to stain the tight junction protein ZO-1, After rapid cell rinsing using 1 × PBS, Caco-2 cells were fixed with 4% paraformaldehyde for 15 min and permeabilized with 0.2% Triton-X 100 for 2 min before incubation with fluorescently labeled ZO-1 antibody at 4 °C for 12 h. Samples were further incubated with antibody for 1 h and counterstained with 4′, 6-diamidino-2-phenylindole (DAPI) for 15 min. After three final washes with PBS, microscopy was performed using a Leica laser confocal microscope.

### Functional characterization of intestinal barrier

On the basis of establishing the morphological integrity of the intestinal microarray barrier, the functional integrity of the human intestinal barrier was assessed by quantifying the specific activity of apical brush border alkaline phosphatase (ALP) and transepithelial electrical resistance (TEER). The specific activity of ALP was determined by obtaining the culture medium from the upper and lower chambers after 5 days of cell culture, transferring the solution to a 96-well plate, and cleaving the product (*e.g.*, 4-nitroaniline) was quantified at 405 nm in a microplate reader. Then, the TEER value was obtained by multiplying the specific resistance which subtracted baseline resistance value (without cells) by the total cell culture surface area on the membrane. DMEM medium served as blank control group.

After establishing tight junction integrity, the apparent permeability coefficient (Papp, cm/s) of intestinal cell monolayers was determined. Papp measurement was achieved primarily by measuring the transit of isosulphate luciferin (FITC)-labeled glucose (FD20, 20 kDa) over time on the intestinal barrier chip. FD20 solution (1 mg/mL) was added to the intestinal barrier on-chip chamber and samples collected hourly from the lower chamber outlet were analyzed to quantify the amount of FD20 that crossed the intestinal barrier. The fluorescence intensity (490 nm excitation/520 nm emission) of the sample collected from the lower chamber was immediately measured to quantify the amount of FD20 transported from the cell apex to the basolateral surface. Papp was calculated according to Papp (cm/s) = (dQ/dt)(1/AC0) where A is the culture surface area (cm^2^), dQ/dt is the steady state flux (g/s) and C0 is the initial concentration (mg/mL) of the FD20 solution applied on the apical cell surface.

### Morphology and distribution analysis

The morphology of the nanofibers was studied by inverted microscope (Olympus IX-71; Olympus, Tokyo, Japan) and field emission scanning electron microscope (SEM, TM3000; Hitachi, Tokyo, Japan). The fibers used in SEM were collected on the conductive adhesive, dried in air, and plated with gold by sputtering gold plating machine (SBC-12; Kyky, Beijing, China) for 60 s (to obtain a gold plating layer of about 10 nm), so as to improve its conductivity. The scanning electron microscope was operated under 15 kV accelerating voltage. Image J software was used to measure the size, orientation angle and distribution of nanofibers (http://rsb.info.nih.gov/ij/).

## Results and Discussion

### Microfluidic device fabrication for aligned nanofibers electrospinning

**Figure 1:**
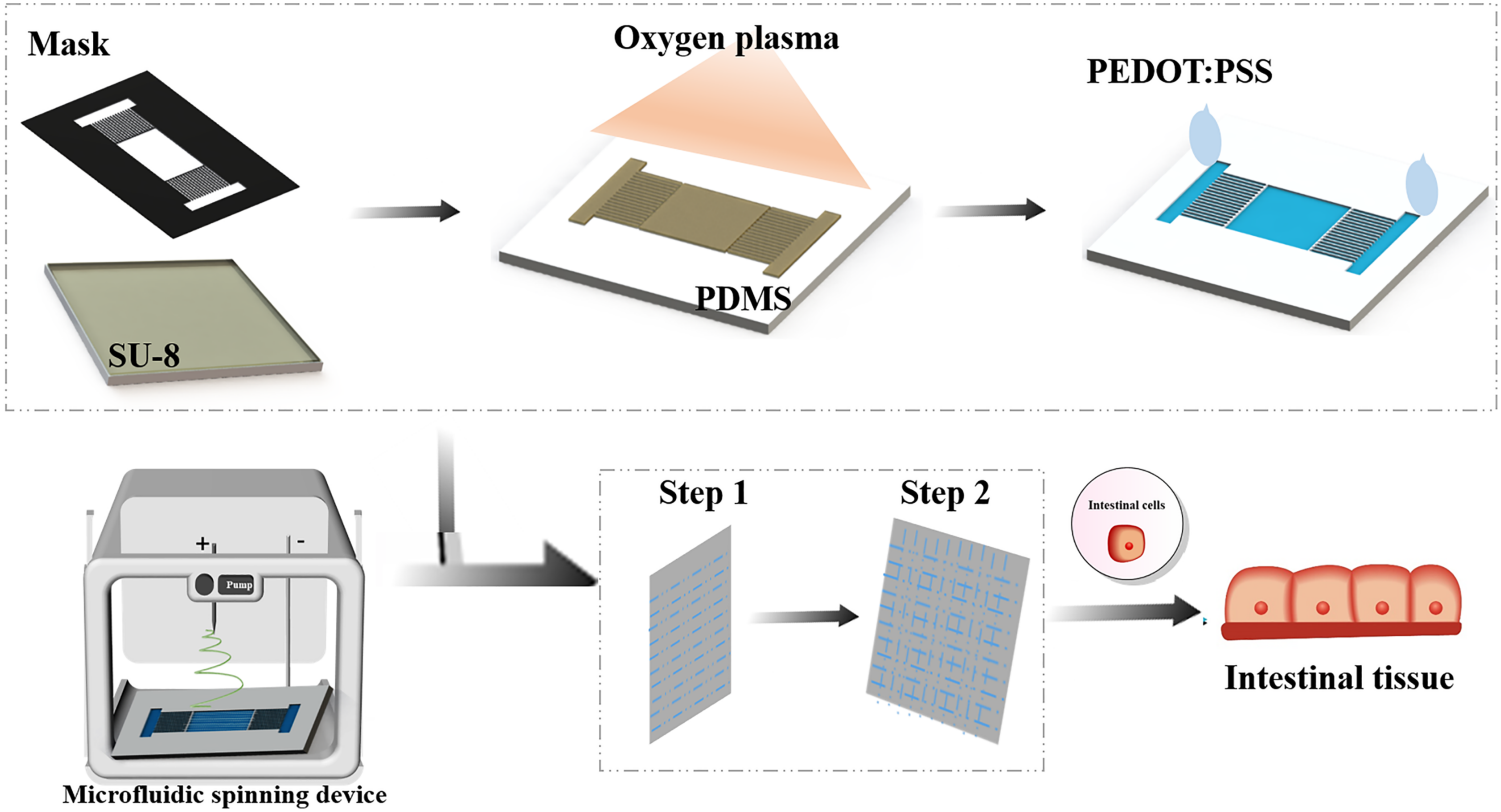
Schematic diagram of the nanofiber scaffold array integrated with microfluidic channels.

Figure 1 shows the sequential steps of fabricating spatially patterned aligned nanofibers on the surface of the dielectric polymer by microfluidic devices. Firstly, the mask pattern with microchannel is generated by laser micromachining, and microfluidic devices are fabricated by mask exposure combined with soft lithography. Secondly, the surface of the microchannel was modified by plasma to obtain a hydrophilic surface. Plasma technology can be compatible with a variety of insulators without changing the overall characteristics of the material so that it can be used for material self-assembly and micro-contact printing. The surface area of the dielectric polymer treated with oxygen plasma becomes more hydrophilic, mainly by introducing oxygen functional groups on the surface of the polymer to increase the surface wettability (Martins et al., 2009). The conductive solution PETO:PSS is added at one end of the microchannel, and the electrolyte solution is selectively positioned on the hydrophilic pattern by virtue of the wetting characteristics of the microchannel (Fig. 1). Then drying for 24 h, which enable to establish the position-selective patterning of the electrolyte solution in microfluidic device.

Next, a high-voltage patterning device is applied between the electrode surface and the metal substrate to control the fluidization. Because the aligned nanofibers are suspended above the microfluidic device, after the nanofibers are collected, they can be easily transferred to the surface of another substrate by moving the collecting electrode vertically for further device processing and manufacturing. An ungrounded carrier made of insulator or metal can also be placed in the gap to directly collect the aligned nanofibers and help prevent the suspended fibers from breaking. Since both ends of the fibers are physically fixed on the surface of the collector, their position and direction can be easily controlled by moving the collector. These fibers can be stacked layer by layer into a multi-layer structure, with the fibers of different layers facing different directions.

**Figure 2:**
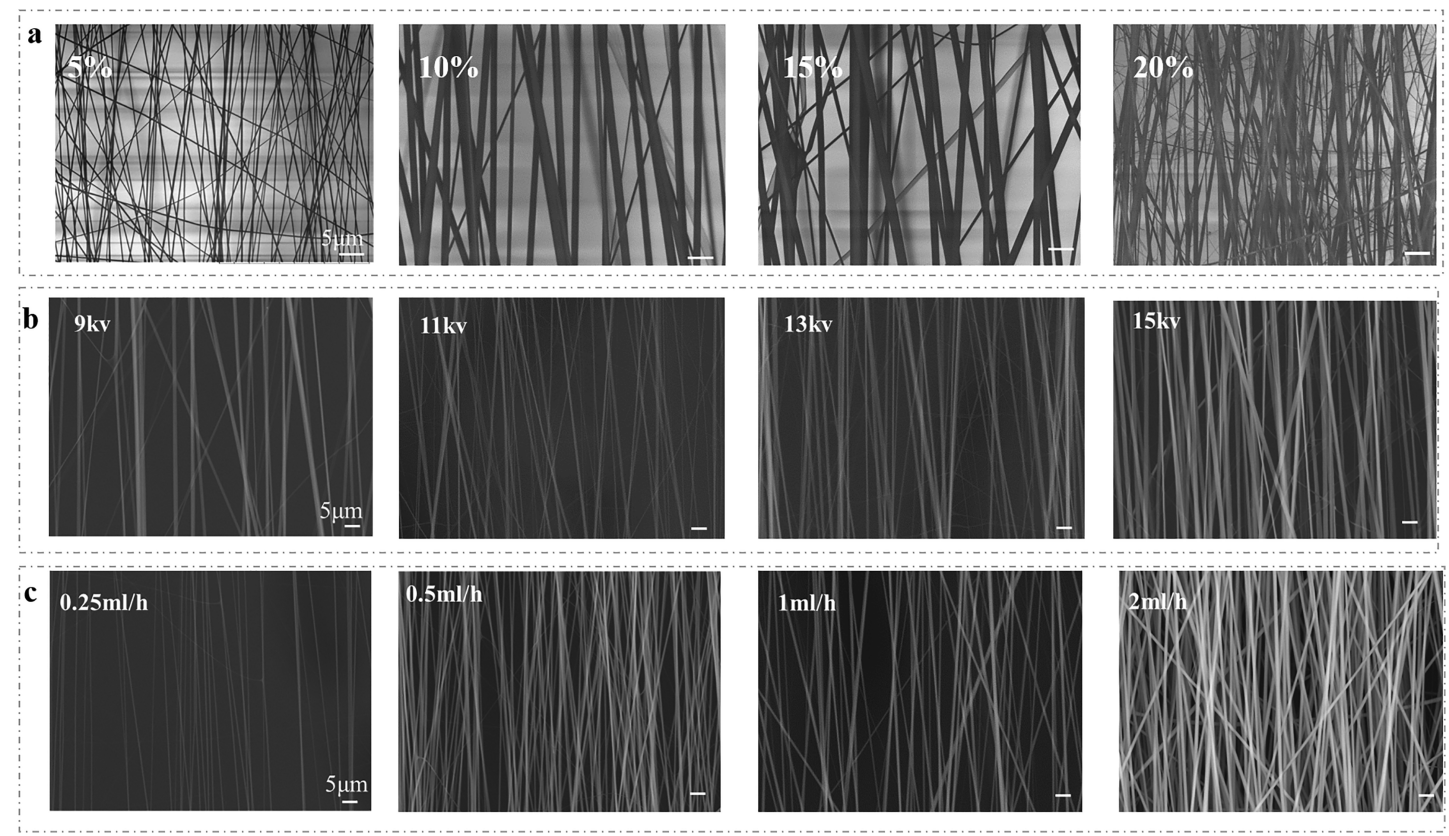
SEM images of nanofibers dissolved in ethanol at various (A) concentration; (B) applied voltages; (C) injection speed.

In order to form the nanofibers regularly, it is needed to find the optimal concentration of the solution. Results as shown in Fig. 2A, PVP nanofibers were prepared under the condition of 11 kV voltage and 0.5 mL/h injection rate. At very low concentration (<3%), the polymer falls onto the surface by electrospray in the form of droplets before being converted into fibers, and the spinning structure cannot be obtained by electron microscopy. The low concentration polymer (5%) solution has low viscoelastic force and the low macromolecular chain entanglement, so the electrostatic repulsion force and coulomb repulsion force needed for spinning cannot match. Although the low concentration polymer is helpful to stretch the electrospun jet, the jet is partially broken, forming irregular curls or spinning fracture. In the case of high tension, a low tension spherical solution is formed on the surface of the polymer. When the solution concentration increases from 5% to 15%, the viscosity increases, resulting in the increase of viscoelasticity and chain entanglement. The increase of solution concentration is related to the production of larger diameter fibers (Doshi & Reneker, 1995).The increase of viscoelastic force prevents the jet from breaking partially, and the solvent molecules are distributed on the entangled polymer molecules, thus forming smooth fibers and improving the uniformity of fibers. When the concentration value is very high (>15%), the fiber becomes very thick, sometimes forms a foil structure, and the spinning diameter becomes more and more uneven, whose phenomenonis consistent with the previous result with high concentration at 21% and 24% (Tiyek et al., 2019). In addition, proper concentration is also a necessary condition to ensure the consistent orientation of nanofibers (Park et al., 2018; Chen et al., 2019; Zhuang et al., 2020).

To form nanofibers, the applied voltage should be sufficient to overcome the surface tension of the solution. Once the electrostatic force overcomes the surface tension, the spinning process begins. Results as shown in Fig. 2B, when the concentration exceeds 10 wt% and the injection speed is 0.5 mL/h, the increase of applied voltage leads to the increase of fiber diameter. The insufficient electric field force in electrospinning process means that the stretching force is inadequate to elongate the solution. When the applied voltage is low (*e.g.*, 9 kV), the Coulomb force is not enough to overcome the surface tension, resulting in the formation of spinning branches. When the applied voltage exceeds a limit voltage (*e.g.*, 20 kV), the thickness of the fiber increases and the irregular structure is formed. The reason is that the Coulomb force is much greater than the viscoelastic force and the jet velocity is much faster. Therefore, there is not enough time for solvent evaporation to form coarse and irregular fibers, which is consistent with the previous results (Baumgarten, 1971). In addition, when the PVP concentration exceeds 10 wt% and the applied voltage exceeds 11 kV, the number of fibers increases sharply with the increase of injection speed (Fig. 2C). It can be seen from Fig. 2C that different injection speeds have little effect on the fiber diameter, only the fiber shape and parallelism, which is consistent with previous reports (Tiyek et al., 2019).

### Microfluidic electrospinning for aligned nanofibers

**Figure 3:**
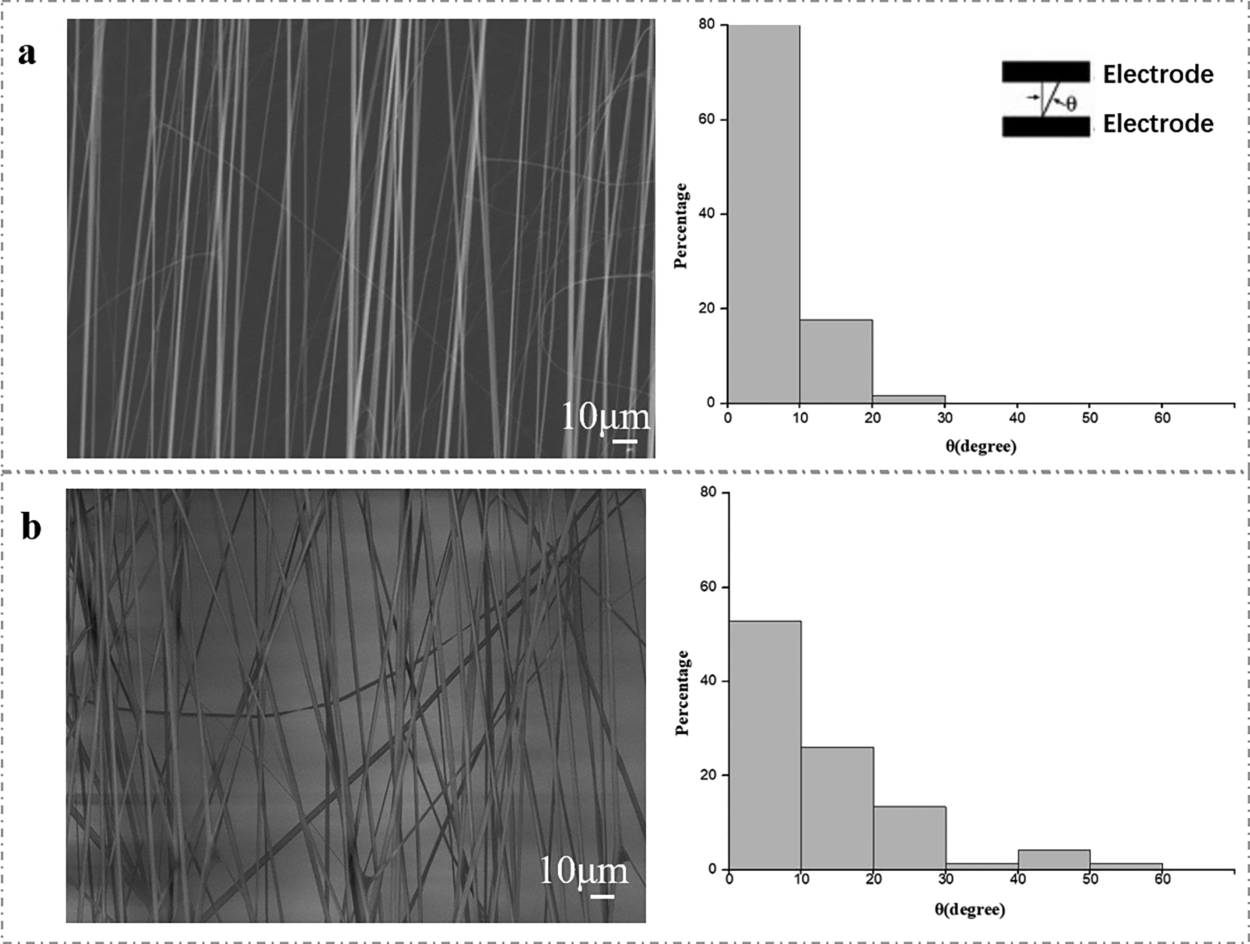
The SEM image of the aligned nanofiber fabricated *via* a microfluidic device (A) and copper foil collectors (B). The SEM image of the aligned nanofiber fabricated *via* a microfluidic device (A) and copper foil collectors (B). The distribution of the normal angle between the long axis of the fiber and the edge of the parallel electrode was also statistically analyzed. The results displayed on each board came from measurements of more than 120 fibers.

The electric field control near the integrated collector can attract highly positively charged electrospun nanofibers to form a patterned or aligned nanofiber mat structure (Park et al., 2018). We compared the effect of microfluidic device and common copper foil on the orientation of nanofibers. The SEM images of PVP electrospun nanofibers are fabricated in the aid of microfluidic patterned electrodes (Fig. 3A) and copper foil electrodes (Fig. 3B). The average diameter of PVP nanofibers produced by microfluidic patterned electrode and copper foil electrode is shown in Fig. 3. Both microfluidic patterned electrodes and copper foil electrodes collector can produce nanofibers, and the morphology of PVP nanofibers will not change with the collector type. The average orientation angle of PVP nanofibers produced by microfluidic patterned electrodes and copper foil electrodes is also shown. Compared with the copper foil electrodes, the rotation angle of the long axis of the nanofibers collected by the microfluidic patterned electrode is smaller, which means that the orientation of the nanofibers is better.

These results indicated that the electrolyte and the metal collector played similar role in the manufacturing process of electrospun nanofibers. In the traditional electrospinning process, the metal collector received the applied high voltage and generated a high-density electric field around the metal needle to facilitate the generation of a charged high-density polymer jet from the Taylor cone of the needle (Li & Xia, 2004a). The microfluidic patterned electrodes in opposite directions can make the positively charged polymer jets be distributed between the electrodes in parallel. During the jetting process of the polymer passing through the needle, especially the electric repulsion between the positive charges within the needle, the polymer jet is reduced to nanometer level (Doshi & Reneker, 1995). The polymer jet undergoes whip like motion induced by the electric repulsion force between positive charges in the jet, which reduces the polymer jet to nanoscale (Liu et al., 2021). Therefore, the microfluidic patterned electrode also allows a continuous process of high charge polymer jet and whipping motion through the repulsive force between charges in the polymer jet through the concentrated electric field at the metal needle.

**Figure 4:**
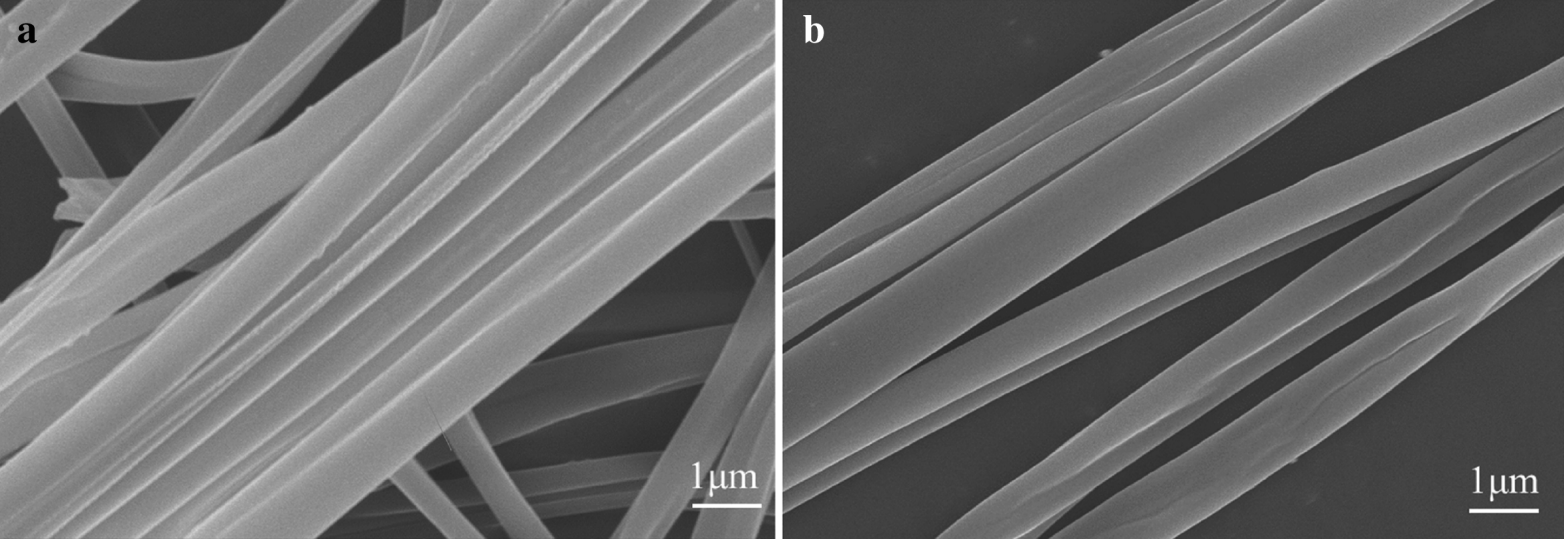
SEM images of uniaxially aligned nanofibers made of different foodborne materials: (A) zein; (B) β–cyclodextrin.

Using the device shown in Fig. 1, we have also successfully prepared uniaxially oriented nanofibers from other food materials, such as cyclodextrin and zein. This method seems to be a general method by which many types of food materials can be electrospun into uniaxially aligned nanofibers without harmful organic solvents. Figure 4 shows an SEM image of the aligned nanofibers with different components and properties (*e.g.*, β-cyclodextrin and zein) with ~500 nm and ~200 nm average fiber diameter, respectively. Edible nanofibers have good biocompatibility and exhibit good proliferative capacity of human hepatocytes and mouse fibroblasts, but are limited to very poor mechanical properties (Dong, Sun & Wang, 2004). By introducing an insulator in the collector, the aligned composite nanofiber array can be easily collected. These fibers can also be directly deposited on glass and PDMS substrates, or transferred to glass and PDMS substrates for calcination or device manufacturing. The combination of nanospinning and template oriented technology can further expand the range of materials that can be made into uniaxially oriented nanofibers (Yang et al., 2007; Ye et al., 2018). The Rayleigh scattering of these food derived nanofiber arrays is also anisotropic, which may be an effective optical polarizer (Li & Xia, 2004b). In addition to the production of nanofibers made from pure organic polymers, biomacromolecules (*e.g*. proteins or enzymes), nanoparticles (*e.g.*, superparamagnetic iron oxide), inorganic nanowires, *etc* (Xue et al., 2017; Alehosseini et al., 2019; Wang, Su & Tan, 2020). These materials can be easily incorporated into electrospinning to obtain uniaxial oriented nanofibers with the required functional combination.

### Multilayered structures of aligned nanofibers on microfluidic devices

**Figure 5:**
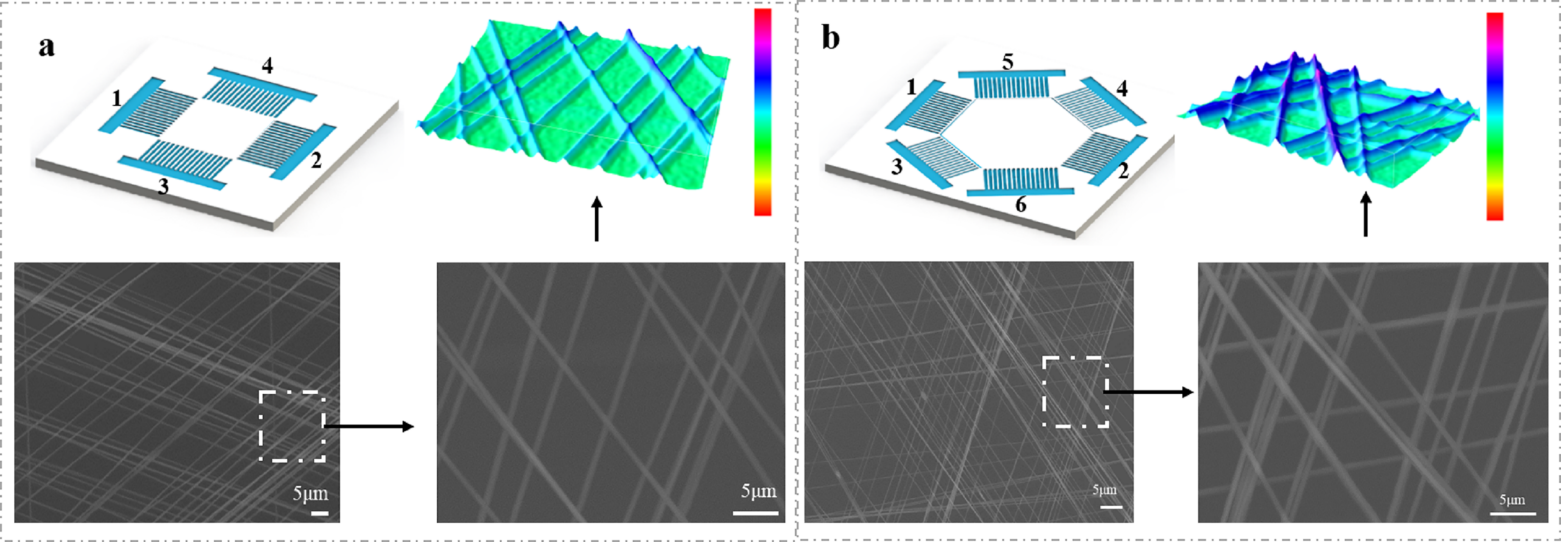
Schematic illustrations of multilayered structures of aligned nanofibers that were composed of four (A) and six (B) electrodes deposited on microfluidic devices. During collection, the electrodes were alternately grounded for ~30 s. The three-dimensional image shows the spatial outline of a grid composed of arranged fibers.

Layers of nanofibers can be stacked in different directions. As shown in Fig. 5, by transferring the uniaxially oriented nanofibers suspended in the gap to the same substrate (in a layer-by-layer manner), the array cross connection can be easily achieved. The aligned nanofibers can be easily stacked into a multilayer structure fiber membrane with a controllable hierarchical structure using a similar method. When multiple pairs of PEDOT:PSS electrodes are used as collectors, PVP nanofibers can also be directly deposited to form a multi-directional parallel fiber array. The spatial orientation and position of PVP nanofibers are determined by the position and structure of the electrodes. By controlling the position of the electrode and applying high voltage, the aligned nanofibers can be easily stacked into multilayer films. By changing the grounding mode of microfluidic patterned electrode pairs *in situ*, the double-layer network structure of nanofibers can be obtained (Fig. 5A). After collecting the first layer on the substrate, we collect the second layer in the electrode direction with 90° rotation angle. Figure 5A shows a SEM image of a rectangular pattern with a rotation angle of 90° formed by the method. The three-dimensional image shows the spatial outline of a grid composed of arranged fibers. We generated a two-layer grid height profile along a straight line through the nanofibers.

The three-layer grid was generated by alternating the grounding electrode pairs (1–4, 2–5 and 3–6) and placing three layers of uniaxially aligned nanofibers with their long axis rotated by 60 degrees. Figure 5B shows the patterning method of microfluidic electrode composed of six gold electrodes. The three-dimensional image shows the spatial outline of a grid consisting of arranged fibers. We generated a three-layer grid height profile through the nanofibers (Fig. 5B). In these cases, the measured angle between the fibers in the different layers is consistent with the corresponding rotation angle. By controlling the electrode structure and/or the sequence of applying high pressure, more composite structures composed of well-aligned nanofibers can be prepared. A remarkable feature of this technology is that in the production process of nanofibers, the arrangement and assembly of nanofibers can be completed simultaneously on the microfluidic device. By using the specially designed electrode as the collector, it is convenient to assemble multi-layer nanofibers with different components on the solid substrate, so as to form a highly ordered layered structure. This kind of structure has potential application value in the manufacture of food, electronic and photonic devices in the future. In addition, different nanofiber arrays can also be integrated into the same device, because a large number of electrode patterns can be read on a single substrate using traditional microfabrication technology.

### Reconstitution of intestinal barrier functions on microfluidic devices

**Figure 6:**
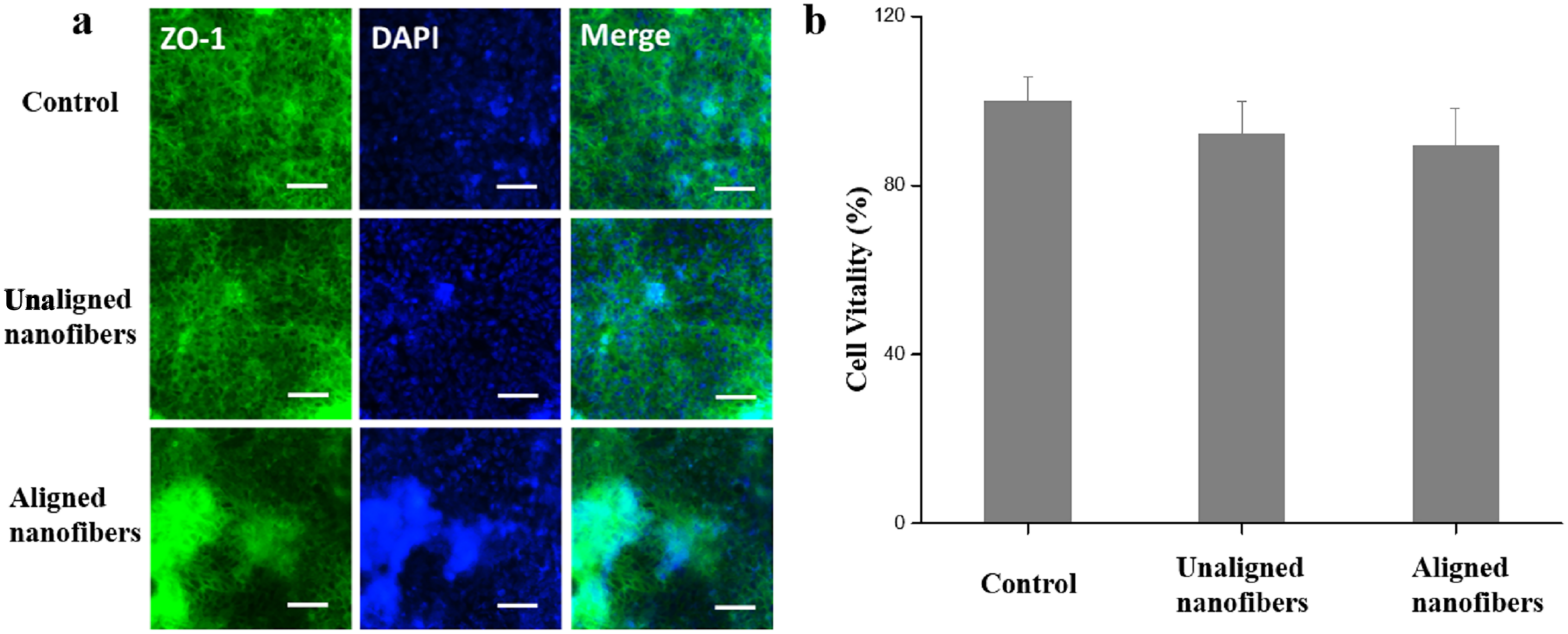
Characterization of intestinal viability of the Caco-2 monolayer under different conditions. (A) Immunofluorescent images show the expression of tight junction protein, ZO-1 (green), in the Caco-2 monolayer. Bars: 50 μm. (B) Quantified analysis of the Caco-2 cell viability by cell counting kit-8 (CCK-8) kit. Data are shown as mean ± SD (*N* = 3).

The chemical properties, surface morphology and structure of biomaterials can affect the function of cells (Lu et al., 2020). To further evaluate the barrier function of the intestinal chip, immunofluorescence microscopes were used to evenly stain the tightly connected protein ZO-1 and to assess the integrity of the tight connection at the top of the intestinal barrier. As shown in Fig. 6A, Caco-2 cells were found formed confluent polygonal epithelial monolayers on nanofibers of microfluidic devices. CCK-8 test also showed PVP non-oriented nanofibers and PVP oriented nanofibers have good biocompatibility and do not affect the viability of Caco-2 cells (Fig. 6B). The Caco-2 cells edges were well delimited by green junctions and well-organized in a characteristic geometry, indicating the integrated tight junction assembly of intestinal Barrier (Liang, Su & Tan, 2021).

**Figure 7:**
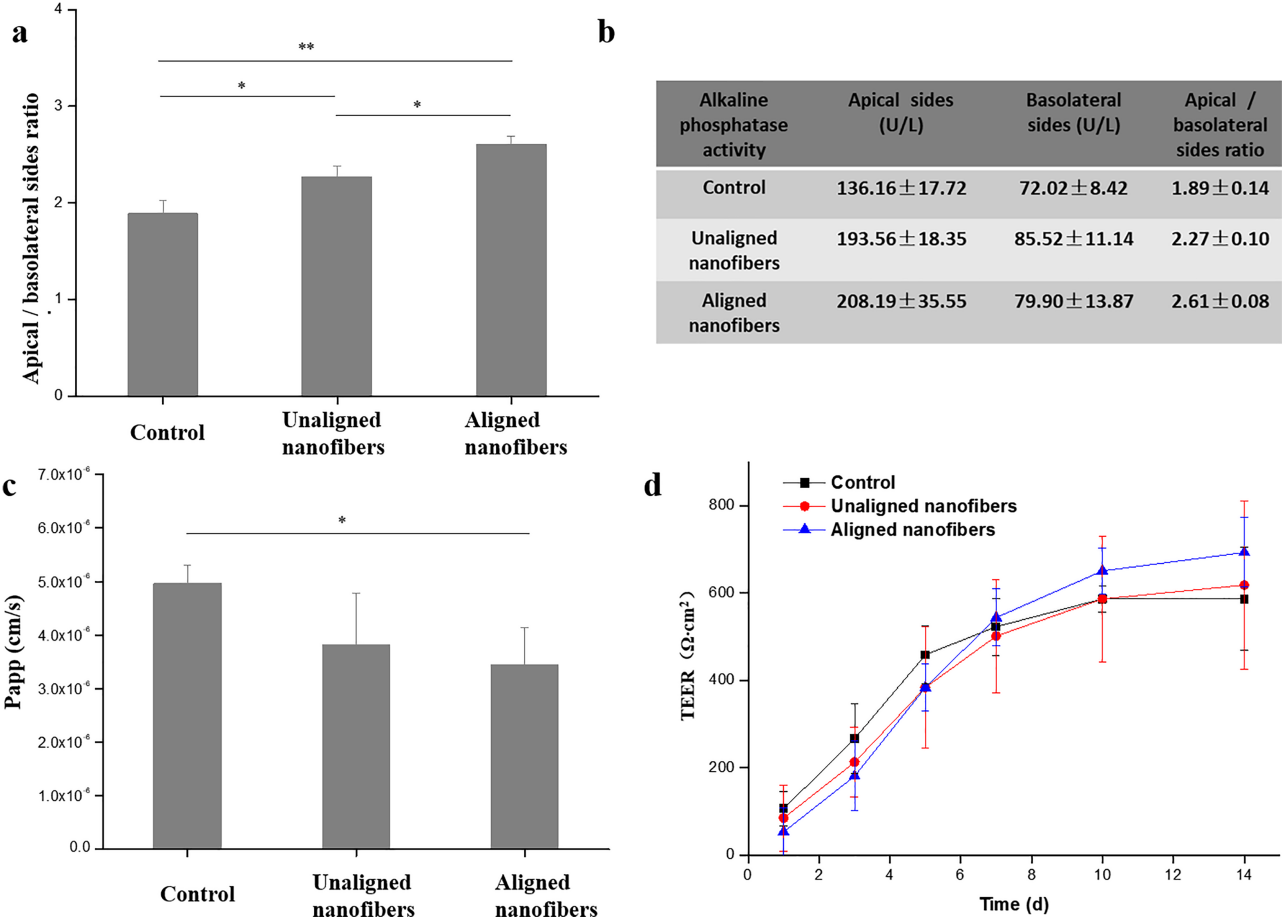
Evaluation of intestinal differentiation and barrier functions under different conditions. (A) The ALP vitality of intestinal cells under distinct cultures were assessed on apical (AP) and basolateral (BL) sides, respectively. Results were obtained from triplicate independent experiments. (B) The apparent permeability coefficient (Papp) was measured by quantifying FD20 transport across the Caco-2 monolayer under different conditions. (C) The barrier integrity of the Caco-2 monolayer was quantified by TEER. Data are shown as mean ± SD (*N* = 3). **P < 0.01, *P < 0.05.

In addition to cytomorphic characterization, we further characterize the intestinal barrier function of the intestinal chip. Alkaline phosphatase (ALP) is commonly used marker for intestinal epithelial cell differentiation as a brush border enzyme. The results showed that cell cultured in the microfluidic device with oriented nanofibers got 2.6-fold higher ALP activity when compared to the cells cultured in control (Figs. 7A, 7B). The results indicated that the oriented nanofibers may promote the differentiation of intestinal cells due to the orderly arrangement structure. Fluorescent glucose was used to measure the Papp value of the intestinal epithelial, so as to characterize the effect of spinning polarity on the intestinal epithelial dysfunction. Compared to the control group, the proliferation of fluorescent dextran in the oriented nanofibers group was significantly reduced due to the presence of intestinal barrier on the chip (Fig. 7C). TEER is an important functional parameter for monitoring the quality of the *in vitro* intestinal barrier. As shown in Fig. 7D, we measured the TEER value of the intestinal barrier chip and subtracted the baseline resistance value of the control sample. The results showed that cells growing under all three cultures showed an increase in TEER values over time during the first 6 days after vaccination, and then maintained similar high levels during at least eight additional days of culture. This small molecule of intestinal filtering barrier selectively as well as the higher TEER levels reproduced the function of intestinal filtration similar to the models *in vivo*.

## Conclusion

In conclusion, we show a simple and effective method to prepare edible micro nanofibers with a large area and suitable arrangement. This method does not require the use of metal collectors and toxic chemicals. Only a small amount of PEDOT:PSS aqueous solution is involved to perform *in situ* spinning within the microfluidic devices. The electrospun fibers are arranged in parallel along the direction of the microfluidic patterned electrode. This method also allows the construction of multidimensional structures based on other complex shapes of parallel arrays (such as meshes). The simplicity of dismantling the collector also enables the electrospun nanofiber membrane to exist independently without any additional steps. The ability of microfluidic devices to produce periodically ordered structures can broaden the application of nanofibers, such as in food manufacturing, nanoelectronic devices, polymer composites and tissue engineering.

## Supplemental Information

10.7717/peerj.13513/supp-1 Supplemental Information 1 Raw data of ALP ratio, CCK-8, Papp, TEER, etc.

## Additional Information and Declarations

### Data Availability

The following information was supplied regarding data availability: The raw measurements are available in the Supplemental File.
